# GWAS with principal component analysis identify QTLs associated with main peanut flavor-related traits

**DOI:** 10.3389/fpls.2023.1204415

**Published:** 2023-09-15

**Authors:** Hui Zhang, Lisa Dean, Ming Li Wang, Phat Dang, Marshall Lamb, Charles Chen

**Affiliations:** ^1^Department of Crop Science and Technology, College of Agriculture, South China Agricultural University, Guangzhou, China; ^2^Department of Crop, Soil, and Environmental Sciences, Auburn University, Auburn, AL, United States; ^3^USDA-ARS Food Science and Market Quality and Handling Research Unit, Raleigh, NC, United States; ^4^US Department of Agriculture-Agricultural Research Service Plant Genetic Resources Conservation, Griffin, GA, United States; ^5^US Department of Agriculture-Agricultural Research Service National Peanut Research Laboratory, Dawson, GA, United States

**Keywords:** flavor, PCA, GWAS, peanuts, QTL

## Abstract

Peanut flavor is a complex and important trait affected by raw material and processing technology owing to its significant impact on consumer preference. In this research, principal component analysis (PCA) on 33 representative traits associated with flavor revealed that total sugars, sucrose, and total tocopherols provided more information related to peanut flavor. Genome-wide association studies (GWAS) using 102 U.S. peanut mini-core accessions were performed to study associations between 12,526 single nucleotide polymorphic (SNP) markers and the three traits. A total of 7 and 22 significant quantitative trait loci (QTLs) were identified to be significantly associated with total sugars and sucrose, respectively. Among these QTLs, four and eight candidate genes for the two traits were mined. In addition, two and five stable QTLs were identified for total sugars and sucrose in both years separately. No significant QTLs were detected for total tocopherols. The results from this research provide useful knowledge about the genetic control of peanut flavor, which will aid in clarifying the molecular mechanisms of flavor research in peanuts.

## 1 Introduction

Peanut, also known as goober or groundnut, is a globally important oilseed crop grown worldwide. Peanuts can be consumed in different formats, such as peanut butter, peanut oil, roasted peanuts, confections, and nutritional bars. The primary and popular means of consumption is roasted peanuts because of their unique, pleasant flavor and abundant nutrients although they can be amenable to various processes (Lykomitros et al., 2016). The improvement of peanut flavor, especially for roasted peanuts, is a long-term goal for the peanut industry. Research has shown that peanut flavor can be affected by genetic, environmental, and storage conditions (Neta et al., 2010). Plants can produce a large variety of flavor volatiles, and the perception of flavor, which includes aroma and taste, is the consequence of different aromatic volatile compound interactions (Zhang et al., 2015).

There are mainly two methods to check peanut flavor including descriptive sensory and analysis by instrument. Analysis by descriptive sensory, which is a forceful tool used to characterize food flavors, makes use of a panel trained in the use of lexicon to evaluate the various flavor characteristics (Meilgaard et al., 1999), while instrumental analysis mainly uses chemical methods to analyze seed composition contents, including moisture content, volatile extracts, tocopherols profile, fatty acids profile, and sugars profile. Moisture content has a significant effect on flavor development reactions. Peanuts have more carbohydrates and glucose in an environment with higher moisture contents (8.7-9.2%) than peanuts stored in an environment with lower humidity (6.2-6.3%) (Pattee et al., 1982). The volatile compositions and concentrations that are present in roasted peanuts determine the flavor although the specific aroma-active compounds responsible for peanut flavor remain elusive (Schirack et al., 2006). All of these seed composition contents can affect peanut flavor; however, the relationship among different contents and which content has the main effect on peanut flavor are still not clear.

The genetics and molecular foundation of flavor, especially volatiles are deficiently understood because of the complexity of flavor/aroma traits. High-throughput sequencing has paved the way for the QTL analysis of aroma components. Genome-wide association studies (GWAS), a powerful tool based on the linkage disequilibrium (LD) theory, are used to detect QTLs associated with complex agronomic traits (Kimani et al., 2020). In GWAS, the fundamental approach is to examine the relationships that exist among each genotyped single-nucleotide polymorphism (SNP) marker and the phenotypes of interest. GWAS does not require the creation of a mapping population, which is in contrast to linkage mapping. At a single locus, they are able to evaluate multiple alleles, and they are also able to offer a higher mapping resolution (Rafalski, 2010; Kimani et al., 2020). QTLs and genes related to tomato flavor have been identified through GWAS (Zhang et al., 2015), and they have been effectively utilized in peanuts to follow hereditary marks related to ionomic (Zhang H. et al., 2019) and agronomic traits (Pandey et al., 2014; Zhang H. et al., 2020), but there is no GWA study in peanut flavor-related traits.

Principal component analysis (PCA) is an effective method to extract a few and major information from complex components and retain most of the variation in the dataset (Ishikawa and Namikawa, 2004; Ringnér, 2008). PCA uses the dimensionality-reduction method to transform multiple correlated phenotypes into a smaller number of uncorrelated variables known as principal components (PCs), which can capture important data points (He et al., 2008). Numerous linkage analyses for correlated traits have utilized it successfully (Karasik et al., 2004; Adeyemo et al., 2005) since this method can decrease the likelihood of type 1 error rate and extract PCs that are closer to the normal distribution (Holberg et al., 2001; He et al., 2008). GWAS and PCA are a good combination when analyzing phenotypically complex traits and this approach has been validated as effective in detecting causal genes related to rice architecture (Yano et al., 2019).

Peanut breeders make the achievement of desirable flavor and quality characteristics as important breeding objective. Various traits can affect peanut flavor, but until now, little information is available on which trait has a bigger effect. Therefore, the aims of the present research were: 1) to explore correlations among moisture, oil content, tocopherols profile, sugars profile, and sensory attributes to identify the main traits that have a major effect on peanut flavor, 2) to determine genomic regions that are associated with main peanut flavor traits, and 3) to identify the candidate genes near the determined QTLs.

## 2 Materials and methods

### 2.1 Plant material and phenotype

#### 2.1.1 Moisture and oil contents

In this experiment, 102 accessions primarily from the U.S. peanut mini-core collection (Holbrook and Dong, 2005) were used (**Table S1**). These genotypes were grown in the field in Dawson, GA, between 2013 and 2014, and each accession with 20 seeds was planted in two-row 10-feet long plots. Prior to growing peanuts, corn-cotton-peanuts were planted on the site (Dothan sandy loam soil) as part of a crop rotation. Gypsum fertilizer was used to grow peanuts in both years in the same quantity (500 pounds/acre). Following the published method, the oil percentage was determined using a Maran pulse nuclear magnetic resonance (NMR, Resonance Instruments, Whitney Oxfordshire, UK) (Wang et al., 2010). The oil percentage was calculated using physiologically mature seeds (∼10 g) on a premise of zero water content by utilizing the equation [oil % * 100/(100 – H_2_O % * 100)]. Seed water contents were estimated, and the mass of the estimation was changed over completely to a percentage of the total weight of each example. All examples were estimated in three replicates.

#### 2.1.2 Tocopherols measurement

A household coffee grinder (Krups International, Frankfurt am Main, Germany) was used to grind the raw peanut samples into a fine meal. Cheesecloth was used to wrap approximately 200 grams of the meal before it was loaded into the X. A hydraulic press (Model 2622-1, Carver, Inc., Wabash, IN) was used to extract the oil. The normal phase HPLC was used to analyze tocopherols in the oil (Hashim et al., 1993). In short, 200 mg of oil was weighed into a 2 mL autosampler vial and then 0.8 mL of hexane was added which contains 2% (v/v) isopropanol. The items in the vial were vortexed to blend and infused onto the HPLC (Agilent Model 110, Agilent Technologies, Santa Clara, CA). The mobile phase was adding 2% (v/v) isopropanol in hexane at 1.2 mL/min in an isocratic manner. The column was a Luna Silica, which was kept at a temperature of 30°C. UV was used for detection at 295nm. Hexane solutions of genuine tocopherols were tested simultaneously as standards. Sigma Chemical Corp. (St. Louise, MO) provided the standards of α, γ, and δ tocopherols, and Matreya LLC (State College, PA) provided β-tocopherol. Retention times and external standards for calculating tocopherol content were determined using standards. Triplicates were used for each analysis.

#### 2.1.3 Sugars profile

A Soxhlet apparatus was used to boil hexane to completely defat the recovered pressed paste for tocopherols. The remaining meal was broken down for sugars analysis as previously depicted (Pattee et al., 2000). In short, approximately 100 mg of defatted peanut meal was extracted with 15 mL of an aqueous solvent mix (chloroform/methanol/water 60/25/15 v/v/v). To extract approximately 100 mg of defatted peanut meal, 15 mL of an aqueous solvent mix was used. In order to pellet the remaining solid material, the samples were then centrifuged at 1000 rpm. In a vacuum oven with a solvent trap, the solvent layer was decanted into a small beaker and evaporated overnight. The evaporated buildup was carried with 1 ml of water containing 2.3 mM lactose and 1.2 mM cellobiose as inside principles. To get rid of free amino acids, the solution was diluted 40 times with water and filtered through a sulfonic acid column (Dionex On Guard IIH, Thermo-Dionex, Sunnyvale, CA). Then, HPLC with an ion exchange column and a pulsed amperometric detector (PAD) was used to analyze the solutions. A Dionex BioLC system with a Dionex PA-1 column (250 mm X 4.6 mm i.d.) and a detector using electrochemistry was used in the HPLC. At 1.0 mL/min, 200 mM sodium hydroxide in water served as the mobile phase. The segment was warmed to 30°C. For quantitation, a standard blend containing the interior principles of myo-inositol, glucose, fructose, sucrose, raffinose, and stachyose was utilized. Sigma Chemical Corp. (St. Louis, MO) supplied all of the standards. Each analysis was performed three times. Reaction factors in light of the inner guidelines were determined and utilized for the quantitation.

#### 2.1.4 Sensory analysis

After harvest, the crude peanuts were first dried and precisely shelled at the USDA, ARS Public Nut Research facility in Dawson, GA. For analysis, the mature peanuts, which had been dried and shelled, were sent to the USDA-ARS Market Quality and Handling Research Unit in Raleigh, North Carolina. Using a colorimeter (Hunter Labs, Reston, VA), the raw peanut samples were roasted in a forced air oven (Model LXD, Despatch Industries, Minneapolis, MN) at 171°C until they reached a final color of L=48 ± 1 on the Hunter scale. A Robot Coupe Blixer 3 commercial food processor (Robot Coupe U.S.A., Inc., Ridgeland, MS) was used to ground samples to a paste. Any variation between seeds was eliminated by this preparation (Sanders et al., 1989).

An eight-member panel that was trained in the descriptive evaluation of peanut flavor and maintained by the USDA-ARS Market Quality and Handling Research Unit in Raleigh, North Carolina, received the pastes. The Spectrum® Flavor Descriptive Analysis Technique was taught to the panel (Meilgaard et al., 1999). Three-digit numbers were used to randomly present the samples. On a 15-point scale, the previously described flavor descriptors were evaluated (Sanders et al., 1989; Johnsen et al., 2004). The examples were introduced aimlessly in copy and scores were accounted as means.

#### 2.1.5 Statistic analysis

Using SPSS Statistics Version 24 (IBM SPSS, IBM Corp, Armonk, NY, USA), the phenotypic data were subjected to statistical analysis. SPSS factor dimension reduction was used in the PCA analysis. The analysis of variance components was assessed utilizing the restricted maximum likelihood (REML) method, and the standard GLM method was used to do the univariate variance analyses. The broad-sense heritability for every trait (with the exception of sensory analysis) across the two environment experiments was determined based on the estimated variance components with the accompanying equation: $hB2=\sigma_{\text{ g}}^{2}/\left(\sigma_{\text{ g}}^{2}+{\text{σ}}_{\text{ g}\times \text{e}}^{2}/\text{r}+{\text{σ}}_{\text{ error}}^{2}/\text{rn}\right)$ , where $\sigma_{\text{ g}}^{2}$ , ${\text{σ}}_{\text{ g}\times \text{e}}^{2}$ , and ${\text{σ}}_{\text{ error}}^{2}$ represent the genotypic variance, the interaction between 102 genotypes and environment variance, and the error (residual) variance component, respectively. r is the number of environment trials and n is the number of replications in each environment trial (Holland et al., 2003).

### 2.2 DNA extraction and genotyping

For DNA extraction, field-grown plant leaves were collected and stored at -80°C. The genomic DNA was extracted using a modified CTAB method (Porebski et al., 1997). For the purpose of analysis, the purified DNA was dissolved in TE buffer. The Nanodrop 2000 was used to measure the DNA’s quantity and quality.

At GeneSeek (Lincoln, Nebraska, USA), the SNP array (Affymetrix 2) was used for the genotyping. No samples were excluded for low call rate (< 0.95) or low quality. After removing SNPs with genotyping error, a call rate of less than 0.95, or a minor allele frequency of less than 0.05, a total of 12,526 SNP markers were retained. STRUCTURE 2.2.3 was used to analyze the population structure of the 102 accessions (Pritchard et al., 2000). In a previous study, the markers’ density and heterozygosity were reported (Zhang H. et al., 2019).

### 2.3 Linkage disequilibrium estimation

The filtered SNP data was used to calculate the linkage disequilibrium (LD) with the PopLDDecay program (Zhang C. et al., 2019). Standardized disequilibrium coefficients (D’) and squared correlations (r^2^) were figured. Based on the means of distances between SNP pairs and r^2^ values, LD decay plots were created.

### 2.4 Genome-wide association studies

To detect the association between SNPs and flavor-related traits in peanuts, statistical analyses were performed using three different methods: (1) TASSEL 5.0: the PCA (first five PCs) + GLM module (general linear model, GLM) was utilized in the TASSEL software (Bradbury et al., 2007). (2) GAPIT 3.0: association mapping was implemented in R utilizing the MLM module (mixed linear model), Y = Xβ + Zu + e (Lipka et al., 2012). (3) BLUP analysis with mrMLM software (Zhang YW et al., 2020).

The TASSEL module matched the data better than the MLM module, as shown by the Q-Q plots. The TASSEL was used to analyze the phenotype and genotype data. LD pruning was accomplished by PLINK (Purcell et al., 2007) using the indep-pairwise function with a step size of 50, a variant count of 5, and r^2^ threshold of 0.5, which generates 2,363 independent SNPs and LD blocks for this population. Based on the number of independent SNPs and LD blocks, the P-value threshold for significant and suggestive QTLs was calculated (Zhou et al., 2017). The LD block was defined as a collection of adjacent SNPs with a pairwise r^2^ value of at least 0.50 (Gu et al., 2011). A total of 2,363 independent SNPs and LD blocks were retained after LD pruning. Thus, the threshold for significant QTLs was 0.05/2363 = 2.12e−5 with − log10 ^(P value)^ = 4.67. Major or significant QTLs were defined as QTLs that met the significant threshold. In a genome-wide test, “suggestive association” permits one false-positive effect, indicating that the threshold p-value was 1/2363 = 4.23e−4 with − log10 ^(P value)^ = 3.37. Suggestive QTLs were defined as QTLs that were lower than the significant threshold but up to the suggestive threshold. Using qqman, Manhattan plots of the p-value results were created (Turner, 2014).

### 2.5 Candidate genes

Genes within the ±80 kb region of the significant and suggestive SNPs associated with peanut flavor were detected from the Tifrunner genome sequences (https://peanutbase.org/). The Tifrunner genome annotation file was used to annotate the identified genes. RNA-Seq data under BioProject PRJNA573070 were downloaded from the NCBI Sequence Read Archive (SRA) database and used for differential expressed genes (DEGs) analysis. Four seed developmental stages (yellow, orange, brown, and black) of four genotypes (AABB, aaBB, AAbb, and aabb) of F8 breeding lines were used for RNA-Seq. For details, see the paper by Zhang et al. (2021).

## 3 Results

### 3.1 Phenotypic analysis by PCA

A total of 102 U.S. peanut mini-core accessions planted in 2013 and 2014 were used in this research. Thirty-three traits associated with flavor, which includes moisture, oil content, tocopherol (total tocopherols, alpha-tocopherol, beta-tocopherol, gamma-tocopherol, and delta-tocopherol), sugars (inositol, glucose, fructose, sucrose, raffinose, stachyose, and total sugars), and descriptive quality (roast peanut, sweet aromatic, raw beany, dark roast, woody hulls skins, earthy musty, cardboardy, plastic chemical, painty, fruity fermented, metallic, spice, sour, sweet, salty, bitter, tongue-throat burn, astringent, and ashy) were measured. Significant variability was identified for these traits among the accessions (**Table 1**). The total sugars varied significantly (ranging from 22502.56 to 40462.04 mcg/g on average 29297.07 mcg/g). Among the total sugars, variation in sucrose ranged from 19999.40 to 37097.90 mcg/g with an average of 25280.23 mcg/g. In addition, the amounts of total tocopherols (293.49-493.25 mcg/g) and gamma-tocopherol (99.38-276.68 mcg/g) also showed significant variation. There is no significant difference in the traits of descriptive quality.

**Table 1:** Variability of flavor-related traits within the U.S. peanut mini-core collection.

Trait	Minimum	Maximum	Mean	SD	hB^2^ heritability (%)	Coefficient of Variation (%)
Moisture (% drywt)	6.41	8.02	7.26	0.28	12.88	3.86
Oil_content (% wetwt)	44.88	53.91	48.15	1.45	77.04	3.01
Alpha-tocopherol (mcg/g fwt)	130.61	269.20	208.60	29.99	86.52	14.38
Beta-tocopherol (mcg/g fwt)	4.61	15.92	7.72	1.82	77.33	23.58
Gamma-tocopherol (mcg/g fwt)	99.38	276.68	174.30	28.12	89.89	16.13
Delta-tocopherol (mcg/g fwt)	4.49	26.98	10.71	3.44	81.62	32.12
Total_tocopherols (mcg/g fwt)	293.49	493.25	401.33	39.87	87.15	9.93
Inositol (mcg/g fwt)	152.86	403.98	222.11	42.19	74.18	19.00
Glucose (mcg/g fwt)	73.17	112.16	85.03	7.65	41.85	9.00
Fructose (mcg/g fwt)	16.25	68.95	31.13	8.69	2.76	27.92
Sucrose (mcg/g fwt)	19999.40	37097.90	25280.23	2851.02	88.76	11.28
Raffinose (mcg/g fwt)	262.76	952.23	458.20	117.08	92.36	25.55
Stachyose (mcg/g fwt)	1546.59	5514.55	3220.37	796.45	78.68	24.73
Total_Sugars (mcg/g fwt)	22502.56	40462.04	29297.07	3199.95	87.47	10.92
Roast_Peanutty	1.96	4.38	3.67	0.41	NA	NA
Sweet_Aromatic	2.06	3.37	2.89	0.27	NA	NA
Dark_Roast	2.08	3.98	3.01	0.33	NA	NA
Raw_Beany	0.13	2.99	2.14	0.45	NA	NA
Woody_Hulls_Skins	2.88	3.58	3.22	0.14	NA	NA
Cardboardy	0.00	1.19	0.31	0.25	NA	NA
Earthy_Musty	0.00	0.53	0.02	0.06	NA	NA
Painty	0.00	0.31	0.01	0.04	NA	NA
Plastic_Chemical	0.00	0.30	0.03	0.07	NA	NA
Metallic	0.00	0.21	0.01	0.03	NA	NA
Fruity_Fermented	0.00	1.06	0.08	0.14	NA	NA
Spice	0.00	1.19	0.05	0.21	NA	NA
Sweet	1.43	3.78	2.76	0.34	NA	NA
Sour	0.00	1.40	0.01	0.14	NA	NA
Salty	0.00	0.00	0.00	0.00	NA	NA
Bitter	1.34	3.73	2.53	0.32	NA	NA
Astringent	1.00	1.47	1.03	0.06	NA	NA
Tongue_Throat_Burn	0.00	0.83	0.05	0.14	NA	NA
Ashy	0.00	2.13	0.55	0.42	NA	NA

Drywt, dry weight; wetwt, wet weight; fwt, fresh weight; SD, standard deviation; hB^2^ = broad-sense heritability estimates obtained by fitting 102 accessions as random terms in the statistical model across growing seasons; NA, it is not possible to get data without replicates.

To investigate the relationships and the main factors underlying trait variation, Pearson correlation and PCA were conducted. For the results of the Pearson correlation, salty has no significant relationship with other traits (**Dataset 1**). PCA results revealed that each PC can only explain a small variance, such as PC1 explaining 18.11% and PC2 explaining 15.32% of total variance (**Table 2A**; **Dataset 2**). This indicated that all of the PCs contributed to total variance without significant difference, so we performed condensed PCA selecting one or two traits from each of the 10 PCs according to the first PCA results. Condensed PCA included 13 traits resulting in five PCs and they explained 68.89% of total variance (**Table 2B**; **Dataset 3**). Considering the normal distribution and component percentage of the phenotype data, three traits (total sugars, sucrose, and total tocopherols) were selected for the following GWAS analysis.

**Table 2A:** Principal component scores of the first 10 PCs for 32 traits (A) and first 5 PCs for 13 traits (B) in the dataset of 102 peanut accessions.

Traits	Components									
	1	2	3	4	5	6	7	8	9	10
Moisture	0.290	0.584	0.301	0.331	-0.223	0.000	-0.062	0.030	-0.137	-0.085
Oil_content	0.058	-0.670	-0.336	-0.232	0.287	0.156	0.155	0.052	0.064	0.159
Alpha	-0.049	0.632	0.308	0.276	0.015	-0.274	0.338	0.073	-0.236	-0.131
Beta	0.158	0.271	0.578	-0.226	0.003	0.103	0.039	-0.340	-0.334	0.240
Gamma	0.121	-0.298	0.637	-0.200	0.438	0.158	-0.044	0.172	0.035	-0.163
Delta	0.146	-0.314	0.654	-0.308	0.162	0.338	-0.161	0.040	-0.061	0.142
Total_tocopherols	0.068	0.251	0.764	0.030	0.334	-0.061	0.211	0.165	-0.173	-0.190
Inositol	0.326	0.223	0.180	-0.148	-0.169	-0.261	-0.016	-0.441	0.393	-0.163
Glucose	0.235	0.372	-0.062	-0.241	-0.501	0.582	0.053	-0.165	0.100	-0.183
Fructose	0.390	0.205	-0.060	-0.243	-0.373	0.614	0.187	-0.123	0.075	-0.154
Sucrose	-0.078	0.882	-0.076	0.075	-0.175	0.025	-0.030	0.215	0.021	0.215
Raffinose	0.578	0.445	0.250	-0.143	0.372	-0.039	0.114	-0.178	0.254	0.133
Stachyose	0.479	0.438	0.131	-0.222	0.428	-0.021	0.055	-0.148	0.261	0.208
Total_Sugars	0.077	0.915	-0.023	0.003	-0.040	0.015	-0.008	0.142	0.099	0.246
Roast_Peanutty	-0.523	0.316	-0.277	-0.152	0.373	0.342	-0.054	0.020	-0.010	0.048
Sweet_Aromatic	-0.567	0.518	-0.299	-0.118	0.267	0.169	-0.011	0.176	0.040	-0.013
Dark_Roast	0.733	0.252	-0.441	-0.044	0.118	0.078	-0.014	0.129	-0.111	-0.021
Raw_Beany	-0.834	-0.209	0.298	0.022	-0.059	0.038	0.065	-0.179	-0.034	0.015
Woody_Hulls_Skins	0.711	-0.110	-0.332	-0.135	0.271	-0.049	0.025	-0.124	-0.208	0.056
Cardboardy	-0.028	-0.406	0.266	0.026	-0.418	-0.087	0.432	-0.178	0.047	0.039
Earthy_Musty	0.309	-0.173	0.385	0.043	-0.278	-0.002	-0.348	0.276	0.190	0.263
Painty	-0.042	-0.198	-0.103	0.022	-0.060	-0.123	0.637	0.156	0.283	0.446
Plastic_Chemical	0.270	-0.288	0.413	-0.025	-0.294	0.292	0.028	0.364	-0.130	0.233
Metallic	0.232	-0.067	0.020	0.057	-0.354	-0.243	-0.502	-0.101	-0.031	0.282
Fruity_Fermented	0.492	0.114	0.074	0.262	0.000	-0.260	0.010	-0.049	-0.055	-0.036
Spice	-0.210	0.034	0.330	-0.034	0.100	-0.093	-0.222	0.195	0.570	-0.250
Sweet	-0.602	0.463	-0.041	-0.294	-0.205	-0.068	0.029	0.188	0.001	0.008
Sour	0.024	0.007	0.010	0.804	0.262	0.355	-0.070	-0.237	0.092	0.158
Bitter	0.793	-0.268	-0.124	-0.250	-0.153	-0.191	0.093	0.208	0.027	-0.108
Astringent	0.171	-0.177	-0.026	0.799	0.140	0.291	-0.025	-0.094	0.082	0.038
Tongue_Throat_Burn	0.417	-0.106	0.042	0.500	-0.098	0.166	0.195	0.324	0.162	-0.192
Ashy	0.844	0.041	-0.291	-0.081	0.139	-0.018	-0.066	0.135	-0.127	-0.116

**Table 2B:** 

Traits	Components				
	1	2	3	4	5
Delta	-0.305	-0.265	-0.327	0.636	-0.171
Total_tocopherols	-0.035	0.202	-0.248	0.730	0.131
Glucose	0.721	-0.451	0.049	0.149	-0.332
Fructose	0.599	-0.582	0.093	0.175	-0.382
Sucrose	0.827	0.439	0.071	0.041	0.241
Total_Sugars	0.836	0.379	0.058	0.117	0.268
Roast_Peanutty	0.243	0.546	-0.097	-0.150	-0.506
Painty	-0.159	0.059	0.025	-0.336	-0.134
Metallic	0.030	-0.321	0.129	-0.122	0.598
Spice	-0.095	0.288	-0.219	0.431	0.187
Sour	-0.152	0.276	0.834	0.277	-0.152
Astringent	-0.257	0.062	0.880	0.247	-0.050
Ashy	0.194	-0.558	0.256	0.045	0.330

### 3.2 Linkage disequilibrium blocks

**Figure 1:**
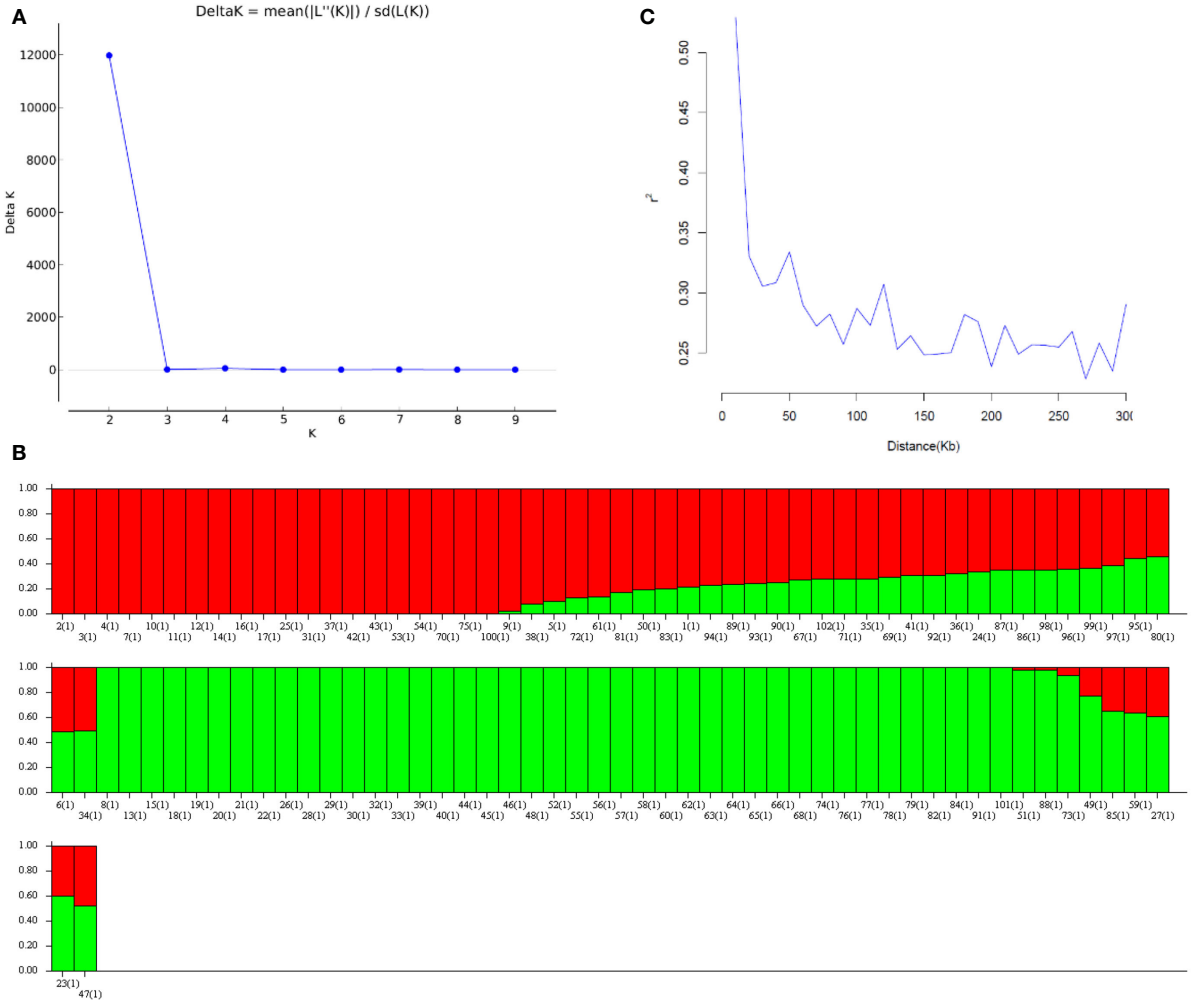
The genetic structure of 102 genotypes mainly comes from the U.S. peanut mini-core collection. **(A)** Δ K information from STRUCTURE analysis of the U.S. peanut mini-core collection. **(B)** Population structure inferred by STRUCTURE analysis. The bar plot for K = 2 was created from 102 accessions and was ordered by Q values. A single vertical line represents each collection and each color represents one cluster. **(C)** Linkage disequilibrium (LD) decade over distance.

After removing SNPs with genotyping error, a call rate of less than 0.95, or a minor allele frequency of less than 0.05, 12,526 SNP markers were retained. No samples were excluded because of low call rates (< 0.95) or poor quality. Population structure results indicated that the 102 mini-core accessions were grouped into primarily two subpopulations according to the delta K (**Figure 1A**), which was in line with our previous study (Zhang H. et al., 2019). Under K = 2, Group 1 contains 52 mini-core genotypes, most of which belong to *hypogaea*, while Group 2 mainly includes *fastigiata* (**Figure 1B**). Linkage disequilibrium (LD) was estimated from r^2^ (r^2^< 0.2 was considered unlinked) against distances between each marker in the 102 U.S. mini-core collections. In this population, the LD decay was approximately 0.16 Mb and the r^2^ was 0.2 (**Figure 1C**).

### 3.3 GWAS for the selected traits

**Figure 2:**
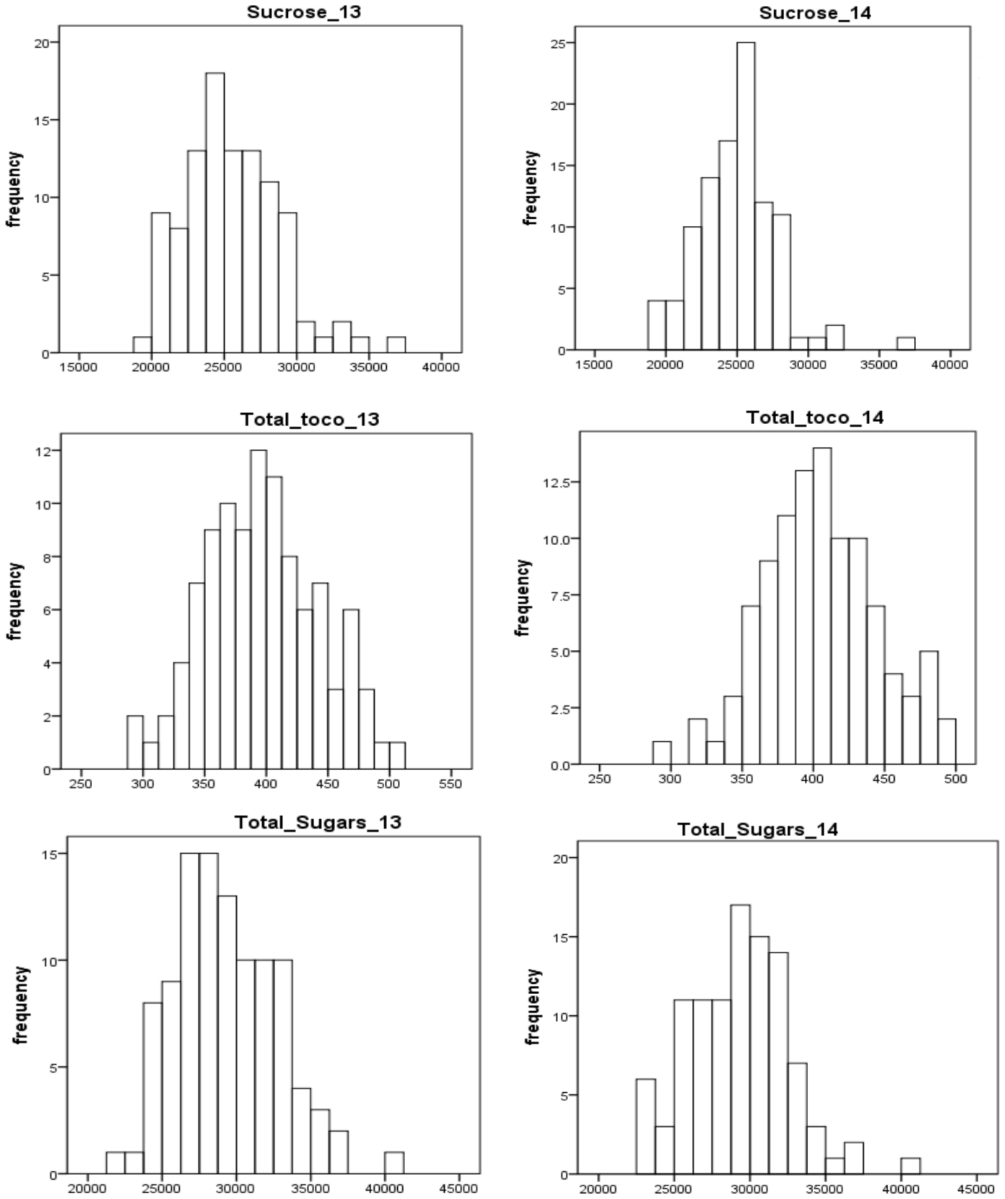
Frequency distribution of total sugars, sucrose, and total tocopherols in peanuts in years 2013 and 2014.

The selected traits (total sugars, sucrose, and total tocopherols) displayed near-normal distribution for both years (**Figure 2**). In general, the distributions of each trait varied little from year to year. For total sugars and sucrose, the ratings ranged from 20000 to 45000 and 15000 to 40000, respectively, in both years 2013 and 2014. For total tocopherols, the ratings ranged from 250 to 550 in 2013 and 250 to 500 in 2014. The median of rating scores for every trait in various years is displayed in **Figure S1**. The estimated broad-sense heritability of total sugars, sucrose, and total tocopherols was 87.47%, 88.76%, and 87.15%, respectively (**Table 1**).

**Figure 3:**
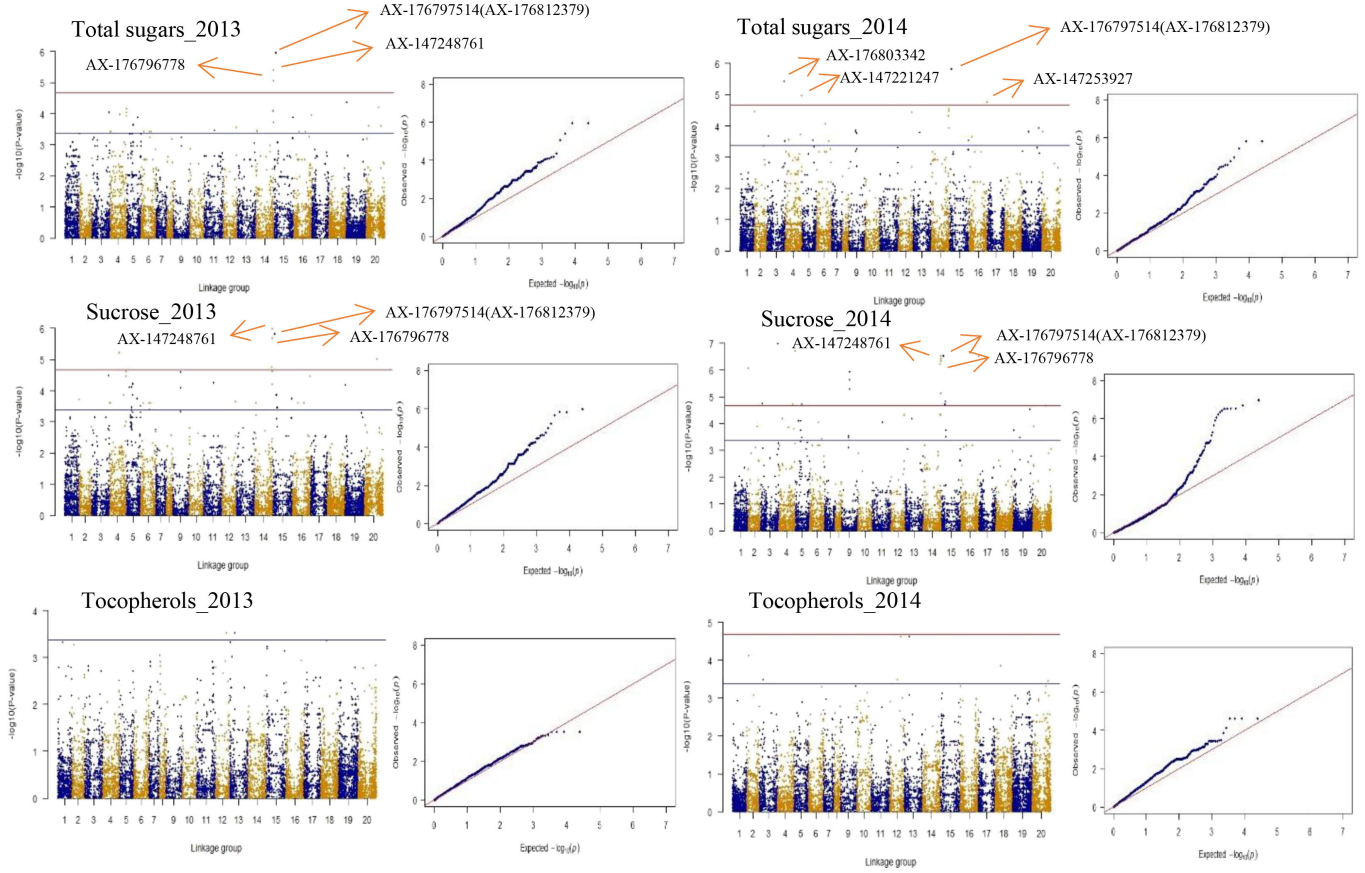
Presentation of Manhattan and Q-Q plots for total sugars, sucrose, and total tocopherols in peanut. The red horizontal line indicates the genome-wide significant threshold: − log_10_^(^*^P^*^value)^ = 4.67. The blue horizontal line indicates the threshold for the significance of “suggestive association”: − log_10_^(^*^P^*^value)^ = 3.37.

**Figure 4:**
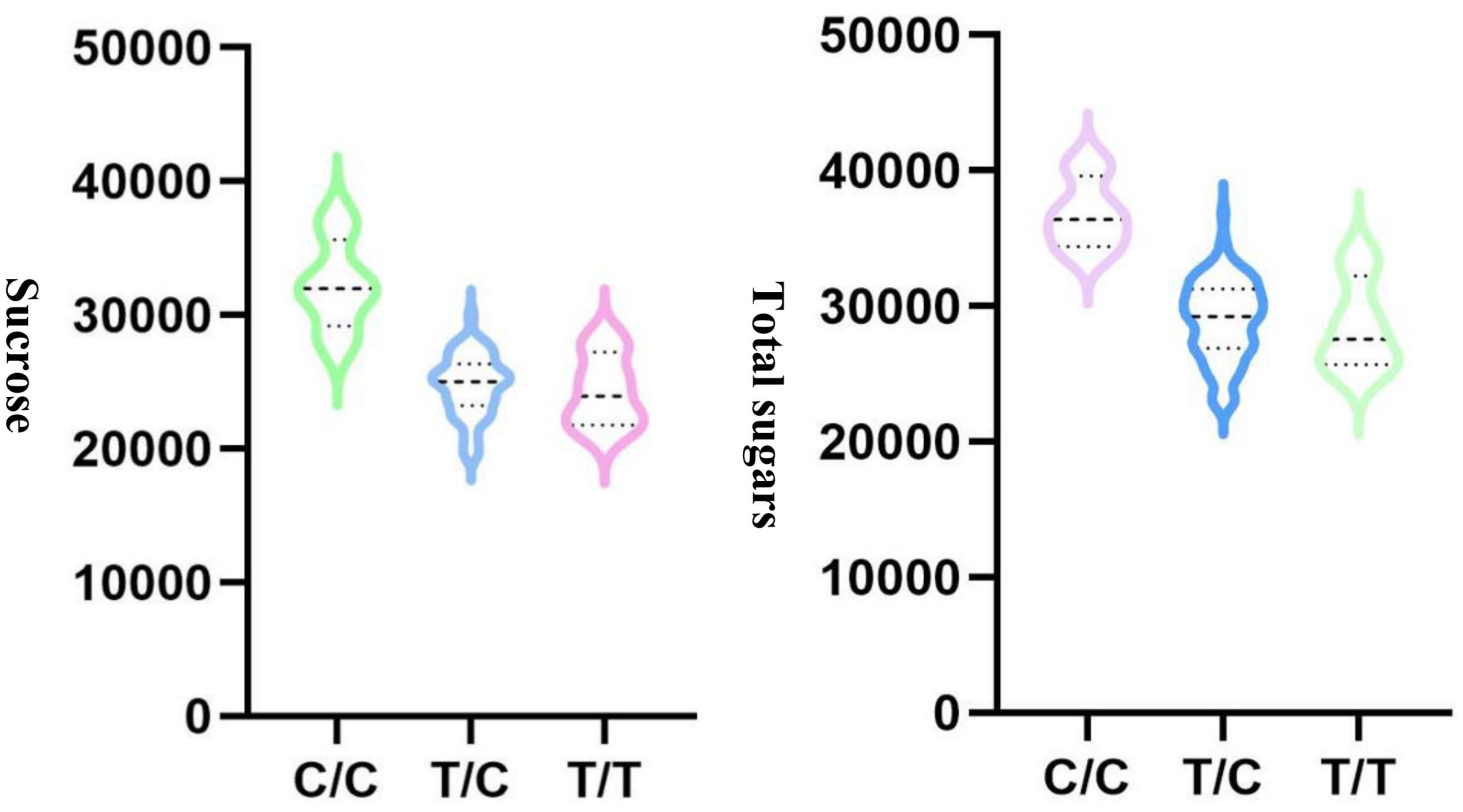
QTL analysis for AX-147221247 located on ChrA04 that is associated with sucrose and total sugars.

To choose the most suitable model for GWAS analysis of the selected traits in the population, the PCA + GLM and MLM models were tested. Quantile-quantile (Q-Q) plots suggested that the PCA + GLM model provided a better fit with the expected distribution (**Figures 3**, **S2**, **S3**). Therefore, the PCA + GLM model was chosen for subsequent analysis. In total, 67, 91, and 20 QTLs were identified for total sugars, sucrose, and total tocopherols, respectively (**Table 3**). Among them, there were seven significant QTLs for total sugars and 22 for sucrose; however, significant QTLs were not identified for total tocopherols. A total of 178 QTLs were found to be distributed in over 15 chromosomes; 81 QTLs were found to be distributed in over six chromosomes of the A sub-genome, and 97 QTLs were found to be mapped in over nine chromosomes of the B sub-genome (**Table S2**). Of the 29 significant QTLs, 12 were situated on the A sub-genome and 17 were on the B sub-genome (**Table S3**). ChrB04 had a maximum of 28 QTLs, followed by ChrA04 with 26 QTLs, and ChrA01, ChrA07, ChrA08, ChrA10, and ChrB07 had no QTLs (**Table S2**). ChrB04 and ChrB05 had the same number of significant QTLs, which is seven (**Table S3**). In addition, two stable significant QTLs (AX-176797514 and AX-176812379) associated with total sugars were identified in both years and both of them explained 16.13% of the phenotypic variance (**Table 4**). Similarly, three stable significant QTLs (AX-147248761, AX-176796778, and AX-147248574) located on ChrB04, and two stable significant QTLs (AX-176797514 and AX-176812379) located on ChrB05 were detected to be related to sucrose. On ChrB05, two significant QTL regions (AX-176797514 and AX-176812379) were found to be related to sucrose and total sugars, respectively. More importantly, QTL AX-147221247 on ChrA04 was significantly associated with sucrose and total sugars. For this QTL, our analysis indicated that the C/C genotype had higher sucrose and total sugars contents, while the T/C or T/T genotype had lower sucrose and total sugars contents (**Figure 4**).

**Table 3:** Summary of QTLs associated with total sugars, sucrose, and total tocopherols in peanuts.

Trait	Year	QTLs identified	Significant QTLs	-log10 (P-Value)	%
Total sugars	2013	40	4	3.42-5.96	9.87-16.13
	2014	29	5	3.52-5.82	7.34-14.05
Sucrose	2013	50	7	3.39-5.99	7.58-15.91
	2014	46	20	3.43-6.96	7.62-17.89
Total tocopherols	2013	4	0	3.52	7.58
	2014	16	0	3.45-4.61	9.13-12.41

% total phenotypic variation explained for SNPs (r^2^×100) on all chromosomes associated with the traits.

**Table 4:** Details of SNPs near significant QTLs identified for total sugars and sucrose.

SNP ID	Trait	Year	Chromosomes	Position (bp)	P-Value	-log10 (P-Value)	%
AX-176797514	Total sugars	2013	B05	17278582	1.10E-06	5.96	16.13
AX-176812379	Total sugars	2013	B05	19291263	1.10E-06	5.96	16.13
AX-147248761	Total sugars	2013	B04	130458494	3.83E-06	5.42	14.96
AX-176796778	Total sugars	2013	B04	130395538	8.67E-06	5.06	14.05
AX-176797514	Total sugars	2014	B05	17278582	1.51E-06	5.82	14.05
AX-176812379	Total sugars	2014	B05	19291263	1.51E-06	5.82	14.05
AX-176803342	Total sugars	2014	A03	126917945	3.68E-06	5.43	13.43
AX-147221247	Total sugars	2014	A04	120249731	1.07E-06	4.97	11.99
AX-147253927	Total sugars	2014	B06	131261613	1.78E-05	4.75	11.78
AX-147248761	Sucrose	2013	B04	130458494	1.03E-06	5.99	15.91
AX-176797514	Sucrose	2013	B05	17278582	1.49E-06	5.83	15.44
AX-176812379	Sucrose	2013	B05	19291263	1.49E-06	5.83	15.44
AX-176796778	Sucrose	2013	B04	130395538	2.13E-06	5.67	15.16
AX-147220124	Sucrose	2013	A04	73767631	5.90E-06	5.23	14.06
AX-176822887	Sucrose	2013	B10	95433617	9.90E-06	5.00	14.14
AX-147248574	Sucrose	2013	B04	127790198	1.77E-06	4.75	13.19
AX-176803342	Sucrose	2014	A03	126917945	1.09E-07	6.96	17.89
AX-147221247	Sucrose	2014	A04	120249731	2.01E-07	6.70	16.83
AX-147248761	Sucrose	2014	B04	130458494	2.93E-07	6.53	17.01
AX-147253927	Sucrose	2014	B06	131261613	3.00E-07	6.52	16.55
AX-176797514	Sucrose	2014	B05	17278582	3.03E-07	6.52	16.54
AX-176812379	Sucrose	2014	B05	19291263	3.03E-07	6.52	16.54
AX-176796778	Sucrose	2014	B04	130395538	3.79E-07	6.42	16.51
AX-147248755	Sucrose	2014	B04	130333319	4.53E-07	6.34	16.94
AX-147248574	Sucrose	2014	B04	127790198	5.87E-07	6.23	15.95
AX-147212613	Sucrose	2014	A02	2117440	8.81E-07	6.06	13.68
AX-147233387	Sucrose	2014	A09	57329521	1.21E-06	5.92	13.52
AX-176791806	Sucrose	2014	A09	57325451	2.27E-06	5.64	15.15
AX-147233383	Sucrose	2014	A09	56931806	5.17E-06	5.29	13.98
AX-147248780	Sucrose	2014	B04	130738535	7.72E-06	5.11	13.57
AX-147249828	Sucrose	2014	B05	32289690	1.47E-05	4.83	13.12
AX-176814258	Sucrose	2014	A03	12720888	1.78E-05	4.75	12.59
AX-176810860	Sucrose	2014	A04	103751547	1.89E-05	4.72	12.53
AX-147222500	Sucrose	2014	A05	50695101	1.89E-05	4.72	12.53
AX-176809744	Sucrose	2014	B05	26697331	1.89E-05	4.72	12.53
AX-176817449	Sucrose	2014	B05	31843948	1.89E-05	4.72	12.53

% total phenotypic variation explained for SNPs (r^2^×100) on all chromosomes associated with the traits.

### 3.4 Genomic regions and genes associated with the selected traits

**Figure 3** depicts the Manhattan plots of the GWAS results. The genes near significant and suggestive QTLs within ± 80 kb regions are shown in **Table 5**, **Dataset 4**, and **Dataset 5**. A total of four potential candidate genes associated with total sugars near significant QTLs were detected, including one stable gene *arahy.BXRJ0F*, which spans a genomic region between 19,236,308 and 19,239,067 bp on ChrB05 encoding nucleotide-diphospho-sugar transferase superfamily protein (**Table 5**). The functional annotation of the other three genes in major QTL regions incorporates two genes (*arahy.V3ZQ6H* and *arahy.94J2Q8*) that encode UDP-glycosyltransferase and one gene (*arahy.0B2YB3*) that encodes acyl-CoA thioesterase. For sucrose, within the significant associated region, eight genes were found, including nucleotide-diphospho-sugar transferase superfamily protein (*arahy.BXRJ0F*), Gag-pol polyprotein n (*arahy.0KX8XR*), probable polygalacturonase (*arahy.ETW0X5*), acyl-CoA thioesterase (*arahy.0B2YB3*), UDP-glycosyltransferase (*arahy.V3ZQ6H*), UDP-Glycosyltransferase superfamily protein (*arahy.94J2Q8*), ATP-binding/protein serine/threonine kinase (*arahy.8E8ZZ7*), and lysosomal alpha-mannosidase-like (*arahy.X2JSTS*). The stable gene *arahy.BXRJ0F* related to sucrose was also detected in both years. In addition, four genes (*arahy.BXRJ0F*, *arahy.V3ZQ6H*, *arahy.94J2Q8*, and *arahy.0B2YB3*) near significant QTL regions were associated with both total sugars and sucrose, which includes one stable gene (*arahy.BXRJ0F*).

**Table 5:** The potential candidate genes’ association with total sugars and sucrose detected from GWAS.

Traits	Year	SNPs	Chr	Pos	Gene ID	Start	End	Gene function
Total sugars	2013	AX-176812379	B05	19291263	*arahy.BXRJ0F*	19236308	19239067	nucleotide-diphospho-sugar transferase superfamily protein
Total sugars	2014	AX-147221247	A04	120249731	*arahy.0B2YB3*	120281537	120284478	acyl-CoA thioesterase
Total sugars	2014	AX-147221247	A04	120249731	*arahy.V3ZQ6H*	120289043	120292411	UDP-glycosyltransferase
Total sugars	2014	AX-147221247	A04	120249731	*arahy.94J2Q8*	120315438	120323907	UDP-Glycosyltransferase superfamily protein
Total sugars	2014	AX-176812379	B05	19291263	*arahy.BXRJ0F*	19236308	19239067	nucleotide-diphospho-sugar transferase superfamily protein
Sucrose	2013	AX-176812379	B05	19291263	*arahy.BXRJ0F*	19236308	19239067	nucleotide-diphospho-sugar transferase superfamily protein
Sucrose	2013	AX-176822887	B10	95433617	*arahy.0KX8XR*	95462718	95463603	Gag-pol polyprotein n
Sucrose	2014	AX-147212613	A02	2117440	*arahy.ETW0X5*	2104036	2109736	probable polygalacturonase
Sucrose	2014	AX-147221247	A04	120249731	*arahy.0B2YB3*	120281537	120284478	acyl-CoA thioesterase
Sucrose	2014	AX-147221247	A04	120249731	*arahy.V3ZQ6H*	120289043	120292411	UDP-glycosyltransferase
Sucrose	2014	AX-147221247	A04	120249731	*arahy.94J2Q8*	120315438	120323907	UDP-Glycosyltransferase superfamily protein
Sucrose	2014	AX-147222500	A05	50695101	*arahy.8E8ZZ7*	50773444	50777476	ATP-binding/protein serine/threonine kinase
Sucrose	2014	AX-176812379	B05	19291263	*arahy.BXRJ0F*	19236308	19239067	nucleotide-diphospho-sugar transferase superfamily protein
Sucrose	2014	AX-147249828	B05	32289690	*arahy.X2JSTS*	32227854	32240292	lysosomal alpha-mannosidase-like

## 4 Discussion

### 4.1 PCA and Pearson correlation

PCA is a useful method to extract main factors from complex traits that are highly correlated, which covers eigenvectors, standard deviation, and covariance (Karamizadeh et al., 2013). Correlation coefficients were calculated from all 33 investigated traits (**Dataset 1**). Salty was proved to have no significant correlation with other traits. Total sugars and total tocopherols are not significantly correlated with each other; however, both of them have a significantly positive relationship with moisture and a negative relationship with oil content. Moisture and oil contents have a significantly negative relationship with each other. After Pearson correlation calculation, 32 correlated traits were selected to conduct PCA. Each of the traits contributed to a small variance and the first three PCs can only explain 43.91% of phenotype variance during the first PCA. Then, the condensed PCA was performed to reduce the PCs, and finally, three traits (total sugars, sucrose, and total tocopherols) were selected which can explain the most variance of peanut flavor. In addition, for descriptive analysis, astringent, sour, metallic, and roast_peanutty also need to be considered when detecting peanut flavor. From the Pearson correlation and PCA study, we know which traits have bigger effects on flavor.

### 4.2 GWAS

GWAS is an effective method to identify QTLs that are linked to the interest traits. However, many variables can affect the consequences of association mapping, which includes genetic diversity, linkage disequilibrium (LD), population structure and size, density of markers, and errors in phenotyping and genotyping data (Gordon and Finch, 2005). At least two methodologies can be used to validate the reliability of GWAS results including approving the QTLs related to the characteristic in various populations and mutual validation through combining association mapping with genetic mapping in Recombinant Inbred Line (RIL) or F2 groups. Abundant genetic diversity, rapid LD decay within a diverse population, and higher density of markers could increase the mapping resolution. In our study, a total of 102 U.S. peanut mini-core accessions were used, and it covered all peanut botanical varieties (var. *fastigiata*, var. *vulgaris*, var. *hypogaea*, var.*aequatoriana*, var. *peruviana*, and var.*hirsuta*) (**Table S1**). Population structure analysis revealed that although the principal component analysis plot did not show any distinct clusters, the mini-core collections were primarily divided into two subpopulations (**Figure S4**). Population structure, allele frequency, recombination rate, and selection could also influence the precision of GWAS by affecting the LD decay (Liu et al., 2020). In this panel, the LD decay was around 160 kb for the entire genome, which is consistent with a former report (Zhang et al., 2020). GWAS has been extensively utilized in peanut research including different traits, such as ionomic (Zhang H. et al., 2019), leaf spots (Zhang et al., 2020), domestication (Zhang et al., 2017), and yield-related traits (Wang et al., 2019); however, no reports concerning GWAS of peanut flavor-related traits have been reported. The findings from this study will have the potential to be used in the genetic improvement of peanuts, as well as provide important information for flavor research.

### 4.3 Peanut flavor and the identified potential genes

Peanut flavor is a complex agronomic trait influenced by many factors. Peanut seed ingredients including free amino acids, reducing sugars, and amino acids released during protein denaturation are important precursors for flavor development through the Maillard reaction pathway. The scoring test and the hedonic scale method are the two most widely used sensory evaluation methods (Oupadissakoon and Young, 1984). An evaluation sheet for peanuts’ off-flavor and texture was developed by Holaday et al. (Holaday et al., 1964), and Thomas et al. (Thomas et al., 1968) utilized the rank preference test to evaluate peanuts’ quality. Arthur D. Little Co. (Cairncross and Sjöström, 2004) first developed a method for descriptive flavor profile analysis and applied it to foods. Professionally trained panelists were hired to evaluate the peanut flavor by eating peanuts; however, this approach is subjective, time and energy-wasting, and not very accurate. In addition, it is impractical to screen for a variety of flavor chemicals and to select for good flavor by tasting a large number of peanuts. With the rapid advances in molecular breeding tools and QTLs/genes discovery, marker-assisted selection could be used to select peanut with desired flavor.

**Figure 5:**
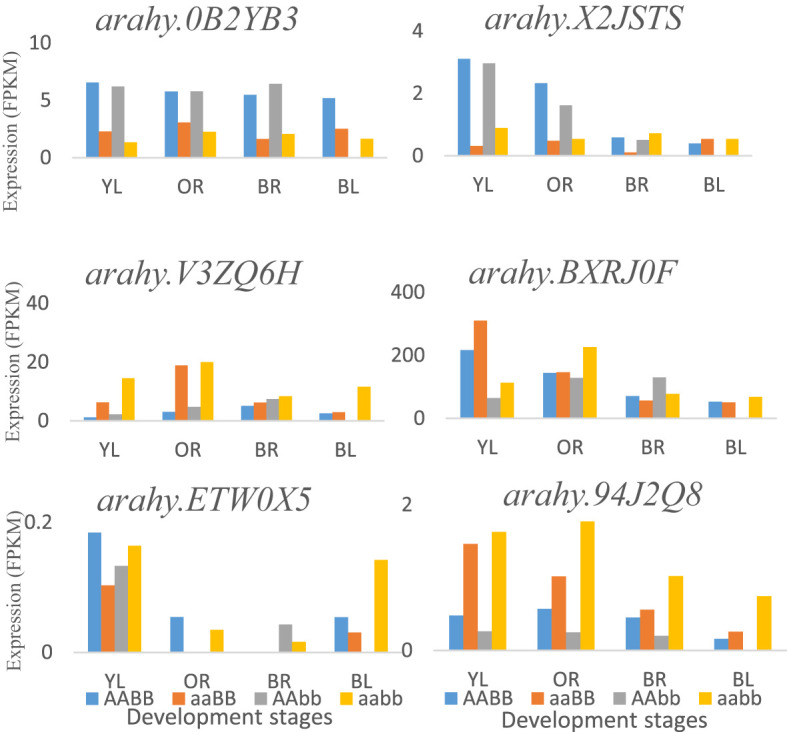
The potential flavor-related DEGs that are around significant QTLs and expressed at different development stages in four peanut genotypes: AABB, aaBB, AAbb, and aabb. YL, Yellow; OR, Orange; BR, Brown; and BL, Black.

By using PCA and GWAS, three important flavor chemicals were detected to have major positive effects on peanut flavor, and sugars are the major contributors to peanut flavor. The related QTLs and genes were obtained, and the function of the potential candidate genes detected from this research is related to the covalent addition and transfer of sugars. Within the genomic region of 160 kb, gene *arahy.V3ZQ6H* (120289043-120292411 bp) and *arahy.94J2Q8* (120315438-120323907 bp), coding for UDP-Glycosyltransferase superfamily, were found to be located on chromosome A04, which catalyzes the covalent addition of sugars from nucleotide UDP-sugar donors to functional groups (amine, hydroxyl, or carboxyl) on an expansive scope of lipophilic molecules (Meech et al., 2019). Glycosyltransferases include multiple gene families that play a role in a process called glycosylation in plant secondary metabolites, which is necessary for the maintenance of cellular homeostasis (Yu et al., 2017). The potential polygalacturonase is encoded by gene *arahy.ETW0X5* plays a role in the biosynthesis of carbohydrates. Some other candidate genes including *arahy.BXRJ0F* (nucleotide-diphospho-sugar transferase superfamily), *arahy.0KX8XR* (Gag-pol polyprotein), *arahy.0B2YB3* (acyl-CoA thioesterase), *arahy.8E8ZZ7* (ATP-binding/protein serine/threonine kinase), and *arahy.X2JSTS* (lysosomal alpha-mannosidase) were found participating in carbohydrate metabolism near significant QTLs regions. In addition, gene expression analysis indicated that most of these genes were involved in the carbohydrate metabolic process and were mainly expressed at the early seed development stages, such as genes *arahy.X2JSTS*, *arahy.BXRJ0F*, *arahy.ETW0X5*, and *arahy.94J2Q8*. There was no significant difference for gene *arahy.0B2YB3* at different seed development stages in four peanut genotypes (**Figure 5**).

Haplotype analysis was conducted for the identified genes; however, no SNPs were detected in the gene regions due to the limitation of the SNP numbers. In this research, the SNPs were from the SNP array, not genome resequencing, which limited the analysis. In addition, the number of accessions also had an influence on the results. To address these problems, whole genome resequencing and multi-environment joint GWAS via 3VmrMLM should be conducted in the future, and the candidate genes should be further confirmed by haplotype analysis and multi-omics analysis.

## 5 Conclusions

To the best of our knowledge, this is the first research on peanut flavor using genome-wide association analysis. The relationships among 33 traits affecting flavor were studied by Pearson correlation. According to our PCA results, three traits (total sugars, sucrose, and total tocopherols) were selected to have major effects on flavor than the other traits, and then GWAS was conducted for these three traits. The use of two-environment, two-year phenotypic data revealed QTL stability across environments. Seven significant QTLs associated with total sugars were identified including two stable QTLs and 22 significant QTLs related to sucrose were detected which also includes two stable QTLs. A total of four promising candidate genes, including *arahy.BXRJ0F*, *arahy.V3ZQ6H*, *arahy.94J2Q8*, and *arahy.0B2YB3* were selected showing that nearby significant QTL regions control both total sugars and sucrose, and *arahy.BXRJ0F* is a stable gene. These findings can provide valuable information for future studies on peanut flavor, and further qTR-PCR or gene function-related experiments will be needed to verify the potential candidate QTLs/genes.

## Data availability statement

The original contributions presented in the study are included in the article/**Supplementary Material**. Further inquiries can be directed to the corresponding authors.

## Author contributions

HZ conducted the statistical analysis and prepared the manuscript. LD collected GWAS data and revised the manuscript. MW and PD prepared the samples and phenotypic data. ML was involved in experiment design and manuscript revising. CC supervised the whole study and provided assistance for manuscript preparation. All authors read and approved the final manuscript.

## References

[B1] Adeyemo A. A. Johnson T. Acheampong J. Oli J. Okafor G. Amoah A.. (2005). A genome wide quantitative trait linkage analysis for serum lipids in type 2 diabetes in an African population. Atherosclerosis 181, 389–397. doi: 10.1016/j.atherosclerosis.2004.12.049 16039295

[B2] Bradbury P. J. Zhang Z. Kroon D. E. Casstevens T. M. Ramdoss Y. Buckler E. S. (2007). TASSEL: software for association mapping of complex traits in diverse samples. Bioinformatics 23, 2633–2635. doi: 10.1093/bioinformatics/btm308 17586829

[B3] Cairncross S. E. Sjöström L. B. (2004). Flavor profiles: a new approach to flavor problems. In Descriptive Sensory Analysis in Practice, M.C. Gacula (Ed.). doi: 10.1002/9780470385036.ch1b

[B4] Gordon D. Finch S. J. (2005). Factors affecting statistical power in the detection of genetic association. J. Clin. Invest. 115, 1408–1418. doi: 10.1172/JCI24756 15931375 PMC1137002

[B5] Gu X. Feng C. Ma L. Song C. Wang Y. Da Y.. (2011). Genome-wide association study of body weight in chicken F2 resource population. PloS One 6, e21872. doi: 10.1371/journal.pone.0021872 21779344 PMC3136483

[B6] Hashim I. B. Koehler P. E. Eitenmiller R. R. (1993). Tocopherols in runner and virginia peanut cultivars at various maturity stages. J. Am. Oil Chem. Soc 70, 633–635. doi: 10.1007/BF02545333

[B7] He L.-N. Liu Y.-J. Xiao P. Zhang L. Guo Y. Yang T.-L.. (2008). Genomewide linkage scan for combined obesity phenotypes using principal component analysis. Ann. Hum. Genet. 72, 319–326. doi: 10.1111/j.1469-1809.2007.00423.x 18261184

[B8] Holaday C. E. Cecil S. Bartlett R. P. (1964). Quality evaluation of mechanically cured peanuts. Proc. Natl. Peanut Res. Conf. 3rd 1964, 91–99.

[B9] Holberg C. J. Halonen M. Solomon S. Graves P. E. Baldini M. Erickson R. P.. (2001). Factor analysis of asthma and atopy traits shows 2 major components, one of which is linked to markers on chromosome 5q. J. Allergy Clin. Immunol. 108, 772–780. doi: 10.1067/mai.2001.119158 11692103

[B10] Holbrook C. C. Dong W. (2005). Development and evaluation of a mini core collection for the US peanut germplasm collection. Crop Sci. 45, 1540–1544. doi: 10.2135/cropsci2004.0368

[B11] Holland J. B. Nyquist W. E. Cervantes-Martínez C. T. (2003). Estimating and interpreting heritability for plant breeding: an update. Plant Breed. Rev. 22, 9–112. doi: 10.1002/9780470650202

[B12] Ishikawa A. Namikawa T. (2004). Mapping major quantitative trait loci for postnatal growth in an intersubspecific backcross between C57BL/6J and Philippine wild mice by using principal component analysis. Genes Genet. Syst. 79, 27–39. doi: 10.1266/ggs.79.27 15056934

[B13] Johnsen P. B. Civille G. V. Vercellott J. R. Sanders T. H. Dus C. A. (2004). Development of a lexicon for the description of peanut flavor. Descr. Sens. Anal. Pract., 533–542. doi: 10.1002/9780470385036.ch6f

[B14] Karamizadeh S. Abdullah S. M. Manaf A. A. Zamani M. HoOman A. (2013). An overview of principal component analysis. J. Signal Inf. Process. 04, 173–175. doi: 10.4236/jsip.2013.43B031

[B15] Karasik D. Cupples L. A. Hannan M. T. Kiel D. P. (2004). Genome screen for a combined bone phenotype using principal component analysis: the Framingham study. Bone 34, 547–556. doi: 10.1016/j.bone.2003.11.017 15003802

[B16] Kimani W. Zhang L.-M. Wu X.-Y. Hao H.-Q. Jing H.-C. (2020). Genome-wide association study reveals that different pathways contribute to grain quality variation in sorghum (Sorghum bicolor). BMC Genomics 21, 112. doi: 10.1186/s12864-020-6538-8 32005168 PMC6995107

[B17] Lipka A. E. Tian F. Wang Q. Peiffer J. Li M. Bradbury P. J.. (2012). GAPIT: genome association and prediction integrated tool. Bioinformatics 28, 2397–2399. doi: 10.1093/bioinformatics/bts444 22796960

[B18] Liu H. Zhan J. Li J. Lu X. Liu J. Wang Y.. (2020). Genome-wide Association Study (GWAS) for Mesocotyl Elongation in Rice (Oryza sativa L.) under Multiple Culture Conditions. Genes 11, 49. doi: 10.3390/genes11010049 PMC7017202 31906181

[B19] Lykomitros D. Fogliano V. Capuano E. (2016). Flavor of roasted peanuts (Arachis hypogaea)-Part I: Effect of raw material and processing technology on flavor, color and fatty acid composition of peanuts. Food Res. Int. 89, 860–869. doi: 10.1016/j.foodres.2016.09.024 28460989

[B20] Meech R. Hu D. G. McKinnon R. A. Mubarokah S. N. Haines A. Z. Nair P. C.. (2019). The UDP-glycosyltransferase (UGT) superfamily: new members, new functions, and novel paradigms. Physiol. Rev. 99, 1153–1222. doi: 10.1152/physrev.00058.2017 30724669

[B21] Meilgaard M. C. Carr B. T. Civille G. V. (1999). Sensory evaluation techniques. (CRC Press. Boca Taton). doi: 10.1201/9781003040729

[B22] Neta E. R. Sanders T. Drake M. A. (2010). Understanding peanut flavor: a current review. Handb. Fruit Veg. Flavors, 985. doi: 10.1002/9780470622834.ch51

[B23] Oupadissakoon C. Young C. T. (1984). Modeling of roasted peanut flavor for some Virginia-type peanuts from amino acid and sugar contents. J. Food Sci. 49, 52–58. doi: 10.1111/j.1365-2621.1984.tb13667.x

[B24] Pandey M. K. Upadhyaya H. D. Rathore A. Vadez V. Sheshshayee M. S. Sriswathi M.. (2014). Genomewide association studies for 50 agronomic traits in peanut using the ‘reference set’ comprising 300 genotypes from 48 countries of the semi-arid tropics of the world. PloS One 9, e105228. doi: 10.1371/journal.pone.0105228 25140620 PMC4139351

[B25] Pattee H. E. Isleib T. G. Giesbrecht F. G. McFeeters R. F. (2000). Investigations into genotypic variations of peanut carbohydrates. J. Agric. Food Chem. 48, 750–756. doi: 10.1021/jf9910739 10725144

[B26] Pattee H. E. Young C. T. Pearson J. L. Singleton J. A. Giesbrecht F. G. (1982). Storage and moisture effects on peanut composition and roasted flavor. Peanut Sci. 9, 98–101. doi: 10.3146/i0095-3679-9-2-14

[B27] Porebski S. Bailey L. G. Baum B. R. (1997). Modification of a CTAB DNA extraction protocol for plants containing high polysaccharide and polyphenol components. Plant Mol. Biol. Rep. 15, 8–15. doi: 10.1007/BF02772108

[B28] Pritchard J. K. Stephens M. Donnelly P. (2000). Inference of population structure using multilocus genotype data. Genetics 155, 945–959. doi: 10.1093/genetics/155.2.945 10835412 PMC1461096

[B29] Purcell S. Neale B. Todd-Brown K. Thomas L. Ferreira M. A. R. Bender D.. (2007). PLINK: A tool set for whole-genome association and population-based linkage analyses. Am. J. Hum. Genet. 81, 559–575. doi: 10.1086/519795 17701901 PMC1950838

[B30] Rafalski J. A. (2010). Association genetics in crop improvement. Curr. Opin. Plant Biol. 13, 174–180. doi: 10.1016/j.pbi.2009.12.004 20089441

[B31] Ringnér M. (2008). What is principal component analysis? Nat. Biotechnol. 26, 303–304. doi: 10.1038/nbt0308-303 18327243

[B32] Sanders T. H. Vercellotti J. R. Crippen K. L. Civille G. V. (1989). Effect of maturity on roast color and descriptive flavor of peanuts. J. Food Sci. 54, 475–477. doi: 10.1111/j.1365-2621.1989.tb03110.x

[B33] Schirack A. V. Drake M. Sanders T. H. Sandeep K. P. (2006). Impact of microwave blanching on the flavor of roasted peanuts. J. Sens. Stud. 21, 428–440. doi: 10.1111/j.1745-459X.2006.00075.x

[B34] Thomas M. C. Lyman C. M. Langley B. C. Senn V. J. (1968). Some factors that affect quality in peanut products as determined by organoleptic evaluation. Food Technol. 22, 108–112.

[B35] Turner S. D. (2014). qqman: an R package for visualizing GWAS results using Q-Q and manhattan plots. bioRxiv, 005165. doi: 10.1101/005165

[B36] Wang M. L. Chen C. Y. Davis J. Guo B. Stalker H. T. Pittman R. N. (2010). Assessment of oil content and fatty acid composition variability in different peanut subspecies and botanical varieties. Plant Genet. Resour. 8, 71–73. doi: 10.1017/S1479262109990177

[B37] Wang J. Yan C. Li Y. Li C. Zhao X. Yuan C.. (2019). GWAS discovery of candidate genes for yield-related traits in peanut and support from earlier QTL mapping studies. Genes 10, 803. doi: 10.3390/genes10100803 31614874 PMC6826990

[B38] Yano K. Morinaka Y. Wang F. Huang P. Takehara S. Hirai T.. (2019). GWAS with principal component analysis identifies a gene comprehensively controlling rice architecture. Proc. Natl. Acad. Sci. 116, 21262–21267. doi: 10.1073/pnas.1904964116 31570620 PMC6800328

[B39] Yu J. Hu F. Dossa K. Wang Z. Ke T. (2017). Genome-wide analysis of UDP-glycosyltransferase super family in Brassica rapa and Brassica oleracea reveals its evolutionary history and functional characterization. BMC Genomics 18, 474. doi: 10.1186/s12864-017-3844-x 28645261 PMC5481917

[B40] Zhang H. Chu Y. Dang P. Tang Y. Jiang T. Clevenger J. P.. (2020). Identification of QTLs for resistance to leaf spots in cultivated peanut (*Arachis hypogaea* L.) through GWAS analysis. Theor. Appl. Genet. 133, 2051–2061. doi: 10.1007/s00122-020-03576-2 32144466

[B41] Zhang C. Dong S.-S. Xu J.-Y. He W.-M. Yang T.-L. (2019). PopLDdecay: a fast and effective tool for linkage disequilibrium decay analysis based on variant call format files. Bioinformatics 35, 1786–1788. doi: 10.1093/bioinformatics/bty875 30321304

[B42] Zhang H. Li Wang M. Dang P. Jiang T. Zhao S. Lamb M.. (2021). Identification of potential QTLs and genes associated with seed composition traits in peanut (Arachis hypogaea L.) using GWAS and RNA-Seq analysis. Gene 769, 145215. doi: 10.1016/j.gene.2020.145215 33038422

[B43] Zhang Y. W. Tamba C. L. Wen Y. J. Li P. Ren W. L. Ni Y. L.. (2020). mrMLM v4. 0.2: an R platform for multi-locus genome-wide association studies. Genom. proteom bioinf. 18 (4), 481–487. doi: 10.1016/j.gpb.2020.06.006 PMC8242264 33346083

[B44] Zhang H. Wang M. L. Schaefer R. Dang P. Jiang T. Chen C. (2019). GWAS and coexpression network reveal ionomic variation in cultivated peanut. J. Agric. Food Chem. 67, 12026–12036. doi: 10.1021/acs.jafc.9b04939 31589432

[B45] Zhang X. Zhang J. He X. Wang Y. Ma X. Yin D. (2017). Genome-wide association study of major agronomic traits related to domestication in peanut. Front. Plant Sci. 8. doi: 10.3389/fpls.2017.01611 PMC5623184 29018458

[B46] Zhang J. Zhao J. Xu Y. Liang J. Chang P. Yan F.. (2015). Genome-wide association mapping for tomato volatiles positively contributing to tomato flavor. Front. Plant Sci. 6, 1042. doi: 10.3389/fpls.2015.01042 26640472 PMC4661238

[B47] Zhou T. Liu S. Geng X. Jin Y. Jiang C. Bao L.. (2017). GWAS analysis of QTL for enteric septicemia of catfish and their involved genes suggest evolutionary conservation of a molecular mechanism of disease resistance. Mol. Genet. Genomics 292, 231–242. doi: 10.1007/s00438-016-1269-x 27826737

