# Supplementary material for: GWAS with principal component analysis identify QTLs associated with main peanut flavor-related traits
Source: Front Plant Sci. 2023 Sep 15;14:1204415. doi: 10.3389/fpls.2023.1204415 (PMC10540862; doi:10.3389/fpls.2023.1204415)
Supplement: Supplementary file 1 [file DataSheet_1.docx]

**Table S1** One hundred and two genotypes mainly coming from the peanut mini core accession used for GWAS analysis related with peanut flavor.

| Code | PI no. | Botanical variety | Origin | Genotype name | Number in structure |
| --- | --- | --- | --- | --- | --- |
| G-001 | PI152146 | *fastigiata* | Uruguay | Turkestan B | 20 |
| G-002 | PI158854 | *fastigiata* | China | N/A | 43 |
| G-003 | PI200441 | *fastigiata* | Japan | Taiwan Kotsubu No. 13 | 8 |
| G-004 | PI259617 | *fastigiata* | Cuba | No. 15233 | 32 |
| G-005 | PI259836 | *fastigiata* | Malawi | Japanese Bunch | 39 |
| G-006 | PI262038 | *fastigiata* | Brazil | R.C.M. 544 | 21 |
| G-007 | PI290566 | *fastigiata* | India | Sl 35 | 2 |
| G-008 | PI290620 | *fastigiata* | Argentina | Sl 89 | 1 |
| G-011 | PI337406 | *fastigiata* | Paraguay | Fav 153 | 13 |
| G-012 | PI339960 | *fastigiata* | Argentina | N/A | 88 |
| G-013 | PI343398 | *fastigiata* | Israel | Fav 153 | 5 |
| G-014 | PI356004 | *fastigiata* | Argentina | Mf.67 XM 27 | 29 |
| G-015 | PI429420 | *fastigiata* | Zimbabwe | Valencia Bassanga | 66 |
| G-016 | PI471954 | *fastigiata* | Zimbabwe | 31/7/28 | 65 |
| G-018 | PI482189 | *fastigiata* | Zimbabwe | Kasawaira | 57 |
| G-019 | PI502040 | *fastigiata* | Peru | SPZ 469-1 | 85 |
| G-020 | PI475863 | *fastigiata* | Bolivia | US 216 | 80 |
| G-021 | PI475918 | *fastigiata* | Bolivia | US 331 | 47 |
| G-023 | PI493329 | *fastigiata* | Argentina | RCM 21 | 60 |
| G-024 | PI493356 | *fastigiata* | Argentina | RCM 48 | 62 |
| G-025 | PI493547 | *fastigiata* | Argentina | RCM 239 | 30 |
| G-026 | PI493581 | *fastigiata* | Argentina | RCM 273 | 74 |
| G-027 | PI493631 | *fastigiata* | Argentina | RCM 323 | 76 |
| G-028 | PI493693 | *fastigiata* | Argentina | RCM 385 | 56 |
| G-030 | PI493729 | *fastigiata* | Argentina | RCM 421 | 77 |
| G-031 | PI493880 | *fastigiata* | Argentina | RCM 572 | 101 |
| G-032 | PI493938 | *fastigiata* | Argentina | RCM 630 | 58 |
| G-033 | PI159786 | *hypogaea* | Senegal | N/A | 14 |
| G-034 | PI162655 | *hypogaea* | Uruguay | 117 | 19 |
| G-035 | PI162857 | *hypogaea* | Sudan | American | 54 |
| G-036 | PI196622 | *hypogaea* | Cote D'Ivoire | 41-48 | 70 |
| G-037 | PI196635 | *hypogaea* | Madagascar | 47-16 | 9 |
| G-038 | PI240560 | *hypogaea* | South Africa | Natal Common | 52 |
| G-039 | PI259658 | *hypogaea* | Cuba | No. 15599 | 31 |
| G-040 | PI259851 | *hypogaea* | Malawi | American | 10 |
| G-041 | PI268586 | *hypogaea* | Zambia | SB41 | 45 |
| G-042 | PI268696 | *hypogaea* | South Africa | BC205 | 15 |
| G-043 | PI268755 | *hypogaea* | Zambia | SB117 | 27 |
| G-044 | PI268806 | *hypogaea* | Zambia | SB152 | 26 |
| G-045 | PI268868 | *hypogaea* | Sudan | BC42 | 17 |
| G-046 | PI268996 | *hypogaea* | Zambia | AR12 | 25 |
| G-047 | PI270786 | *hypogaea* | Zambia | MS. 28 | 28 |
| G-048 | PI270905 | *hypogaea* | Zambia | F1-23 | 24 |
| G-049 | PI270907 | *hypogaea* | Zambia | F1-30 | 23 |
| G-051 | PI290536 | *hypogaea* | India | Sl 5 | 4 |
| G-052 | PI290594 | *hypogaea* | India | Sl 63 | 3 |
| G-053 | PI292950 | *hypogaea* | South Africa | No. 3 | 53 |
| G-054 | PI295250 | *hypogaea* | Israel | Tanganyika Mwitunde | 37 |
| G-056 | PI296550 | *hypogaea* | Israel | 178 | 35 |
| G-057 | PI296558 | *hypogaea* | Israel | 266-4-PL-64 | 36 |
| G-058 | PI298854 | *hypogaea* | South Africa | Jumbo Runner | 16 |
| G-059 | PI319768 | *hypogaea* | Israel | 1066-20 | 34 |
| G-060 | PI323268 | *hypogaea* | Pakistan | No. 45 | 75 |
| G-061 | PI325943 | *hypogaea* | Venezuela | Mani | 40 |
| G-062 | PI331297 | *hypogaea* | Argentina | 132 | 89 |
| G-063 | PI331314 | *hypogaea* | Argentina | 149 | 87 |
| G-064 | PI337293 | *hypogaea* | Brazil | 412 | 22 |
| G-065 | PI337399 | *hypogaea* | Morocco | White Spanish 32 | 73 |
| G-066 | PI343384 | *hypogaea* | Israel | 1318-6 | 6 |
| G-067 | PI355268 | *hypogaea* | Mexico | Almacenado sin cascara | 100 |
| G-068 | PI355271 | *hypogaea* | Mexico | No. 24 | 11 |
| G-069 | PI370331 | *hypogaea* | Israel | Virginia adom | 38 |
| G-070 | PI372271 | *hypogaea* | Unknown | 5009.7 | 12 |
| G-073 | PI442768 | *hypogaea* | Zimbabwe | P34/6/2 | 61 |
| G-074 | PI461434 | *hypogaea* | China | Mao-Ming Hwa-Sung | 68 |
| G-075 | PI471952 | *hypogaea* | Zimbabwe | 31/7/10 | 59 |
| G-076 | PI476636 | *hypogaea* | Nigeria | M 107-74 | 50 |
| G-077 | PI481795 | *hypogaea* | Mozambique | Mative | 64 |
| G-078 | PI482120 | *hypogaea* | Zimbabwe | Kaboko | 63 |
| G-079 | PI494795 | *hypogaea* | Zambia | ZFA 3415 | 102 |
| G-081 | PI496448 | *hypogaea* | Burkina Faso | Kongoussi III | 83 |
| G-082 | PI504614 | *hypogaea* | Colombia | Tatui-76 | 84 |
| G-083 | PI338338 | *peruviana* | Venezuela | Virginia No. 4 | 41 |
| G-085 | PI502120 | *peruviana* | Peru | SPZ 497-1 | 86 |
| G-086 | PI155107 | *vulgaris* | Uruguay | LE 39 Aceitero Federacion | 18 |
| G-087 | PI157542 | *vulgaris* | China | N/A | 42 |
| G-089 | PI271019 | *vulgaris* | Zambia | Spanish 44D 1302 | 44 |
| G-090 | PI288146 | *vulgaris* | India | G 287 | 33 |
| G-092 | PI403813 | *vulgaris* | Argentina | Japones de Tancacha | 46 |
| G-093 | PI407667 | *vulgaris* | Thailand | Chiba Shoryer | 55 |
| G-094 | PI478819 | *vulgaris* | India | Chiba Shoryer | 49 |
| G-095 | PI476432 | *hypogaea* | Nigeria | Chiba Shoryer | 51 |
| G-096 | PI497517 | *fastigiata* | Brazil | US 861 | 82 |
| G-098 | PI497318 | *hypogaea* | Bolivia | US 619 | 81 |
| G-100 | PI494018 | *vulgaris* | Argentina | RCM 710 | 78 |
| G-101 | PI494034 | *vulgaris* | Argentina | RCM 726 | 79 |
| G-102 | PI288210 | *vulgaris* | India | 526 | 90 |
| G-103 | PI371521 | *hypogaea* | Israel | NC25 | 7 |
| G-104 | PI461427 | *hypogaea* | China | Se-li-Hwong | 48 |
| G-105 | Grif12545 | *aequatoriana* | Ecuador | N/A | 91 |
| G-107 | Grif14051 | *aequatoriana* | Guatemala | N/A | 92 |
| G-109 | PI390428 | *hypogaea* | Ecuador | N/A | 67 |
| G-110 | PI468250 | *hypogaea* | Bolivia | N/A | 69 |
| G-111 | PI497648 | *fastigiata* | Ecuador | N/A | 71 |
| G-112 | PI501272 | *hypogeae* | Peru | N/A | 72 |
| G-113 | PI576613 | *hirsuta* | Mexico | N/A | 93 |
| G-114 | PI576614 | *hirsuta* | Mexico | N/A | 94 |
| G-119 | PI648241 | *hirsuta* | Ecuador | N/A | 95 |
| G-120 | PI648242 | *aequatoriana* | Ecuador | N/A | 96 |
| G-121 | PI648245 | *aequatoriana* | Ecuador | N/A | 97 |
| G-122 | PI648249 | *aequatoriana* | Ecuador | N/A | 98 |
| G-123 | PI648250 | *aequatoriana* | Ecuador | N/A | 99 |

Table S2. The distribution of the QTLs associated with total sugar, sucrose, and tocopherols on peanut linkage groups.

| A sub-genome  Linkage Group | Total sugar | Sucrose | Tocopherols | Total |
| --- | --- | --- | --- | --- |
| A01 | 0 | 0 | 0 | 0 |
| A02 | 1 | 3 | 1 | 5 |
| A03 | 4 | 3 | 1 | 8 |
| A04 | 12 | 14 | 0 | 26 |
| A05 | 5 | 16 | 0 | 21 |
| A06 | 5 | 6 | 0 | 11 |
| A07 | 0 | 0 | 0 | 0 |
| A08 | 0 | 0 | 0 | 0 |
| A09 | 3 | 7 | 0 | 10 |
| A10 | 0 | 0 | 0 | 0 |
| B01 | 1 | 2 | 0 | 3 |
| B02 | 2 | 2 | 3 | 7 |
| B03 | 1 | 1 | 6 | 8 |
| B04 | 15 | 13 | 0 | 28 |
| B05 | 6 | 13 | 0 | 19 |
| B06 | 5 | 4 | 0 | 9 |
| B07 | 0 | 0 | 0 | 0 |
| B08 | 0 | 0 | 1 | 1 |
| B09 | 3 | 4 | 0 | 7 |
| B10 | 4 | 3 | 8 | 15 |
| Total | 67 | 91 | 20 | 178 |

Table S3. The distribution of the significant QTLs associated with total sugar, sucrose, and tocopherols on peanut linkage groups.

| A sub-genome  Linkage Group | Total sugar | Sucrose | Tocopherols | Total |
| --- | --- | --- | --- | --- |
| A01 |  |  |  |  |
| A02 |  | 1 |  | 1 |
| A03 | 1 | 2 |  | 3 |
| A04 | 1 | 3 |  | 4 |
| A05 |  | 1 |  | 1 |
| A06 |  |  |  |  |
| A07 |  |  |  |  |
| A08 |  |  |  |  |
| A09 |  | 3 |  | 3 |
| A10 |  |  |  |  |
| B01 |  |  |  |  |
| B02 |  |  |  |  |
| B03 |  |  |  |  |
| B04 | 2 | 5 |  | 7 |
| B05 | 2 | 5 |  | 7 |
| B06 | 1 | 1 |  | 2 |
| B07 |  |  |  |  |
| B08 |  |  |  |  |
| B09 |  |  |  |  |
| B10 |  | 1 |  | 1 |
| Total | 7 | 22 | 0 | 29 |


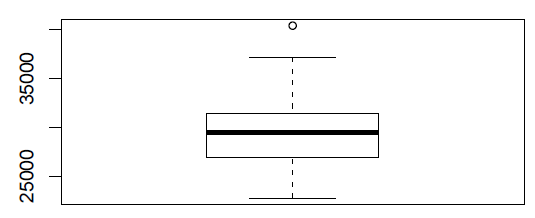

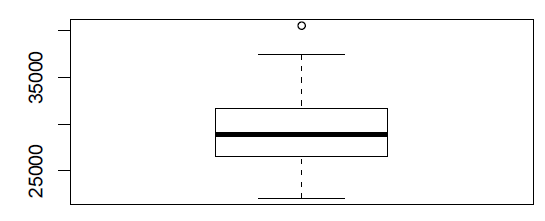


Total sugar14

Total sugar13


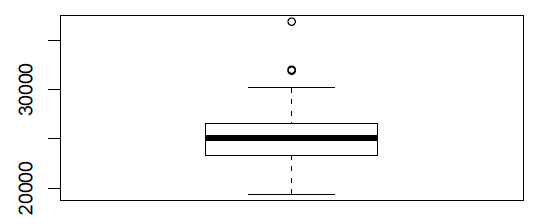

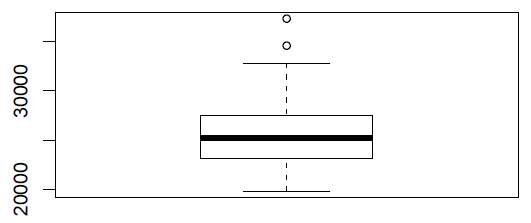


Sucrose14

Sucrose13


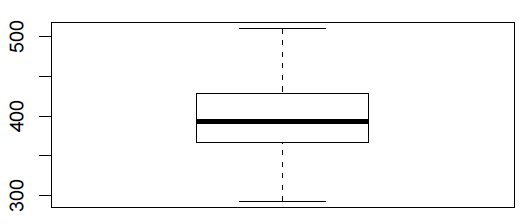


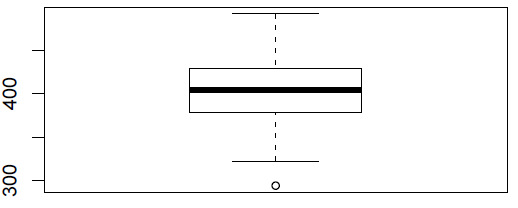


Tocopherols14

Tocopherols13

Fig S1. Average concentrations of total sugar, sucrose, and tocopherols in 102 accessions mainly coming from the U.S. peanut mini core collection in 2013 and 2014. Total sugar, sucrose and tocopherols, mcg/g fresh weight.


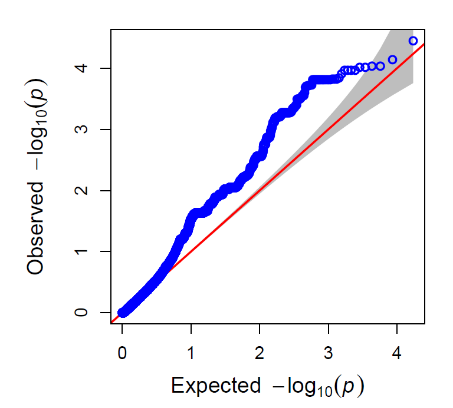

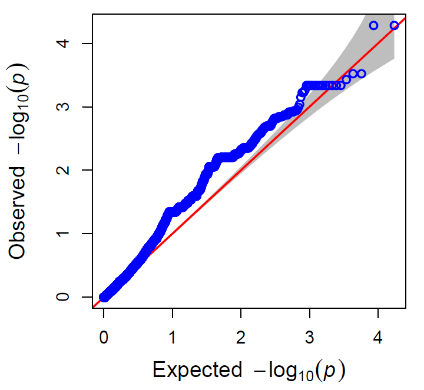


Total sugar_2014

Total sugar_2013


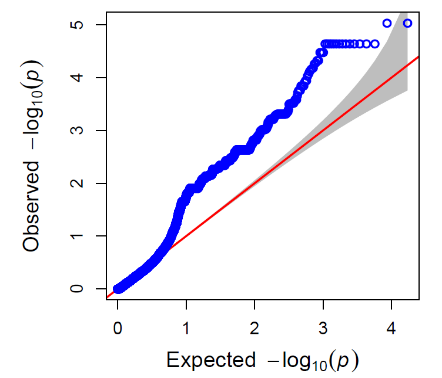

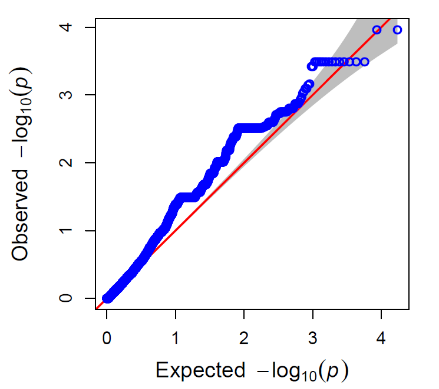


Sucrose_2014

Sucrose_2013


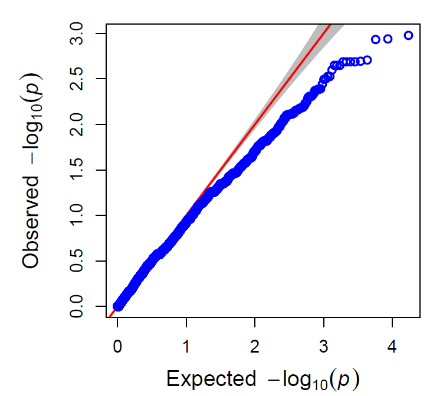

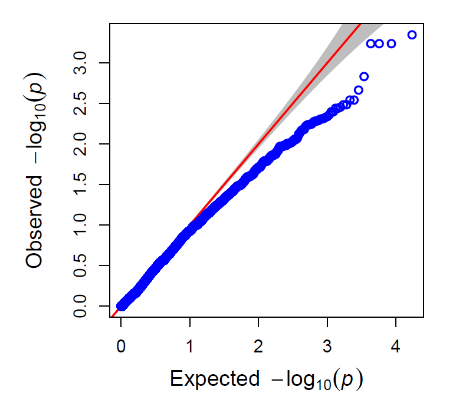


Total tocopherols_2014

Total tocopherols_2013

Fig S2. Q-Q plots for total sugar, sucrose, and total tocopherols resulted from GAPIT. X-axis shows expected − log_10_ ^(^*^P^* ^value)^; y-axis shows observed − log_10_ ^(^*^P^* ^value)^.


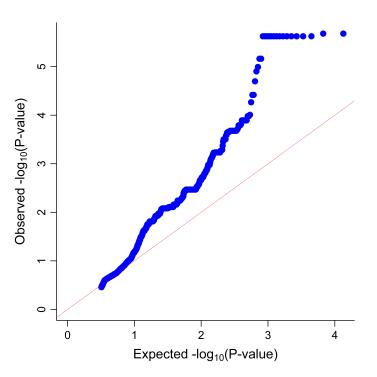

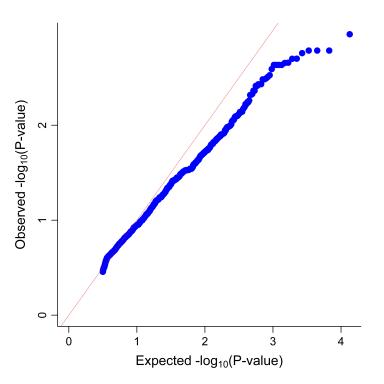

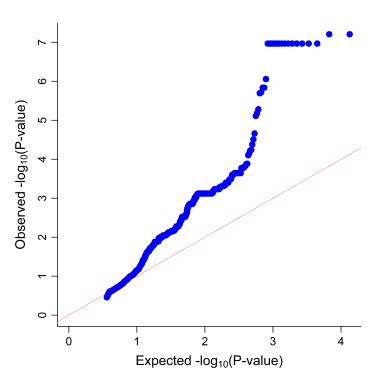


Total sugar

Sucrose

Total tocopherols

Fig S3. Q-Q plots for total sugar, sucrose, and total tocopherols resulted from BLUP analysis with mrMLM software. X-axis shows expected − log_10_ ^(^*^P^* ^value)^; y-axis shows observed − log_10_ ^(^*^P^* ^value)^.


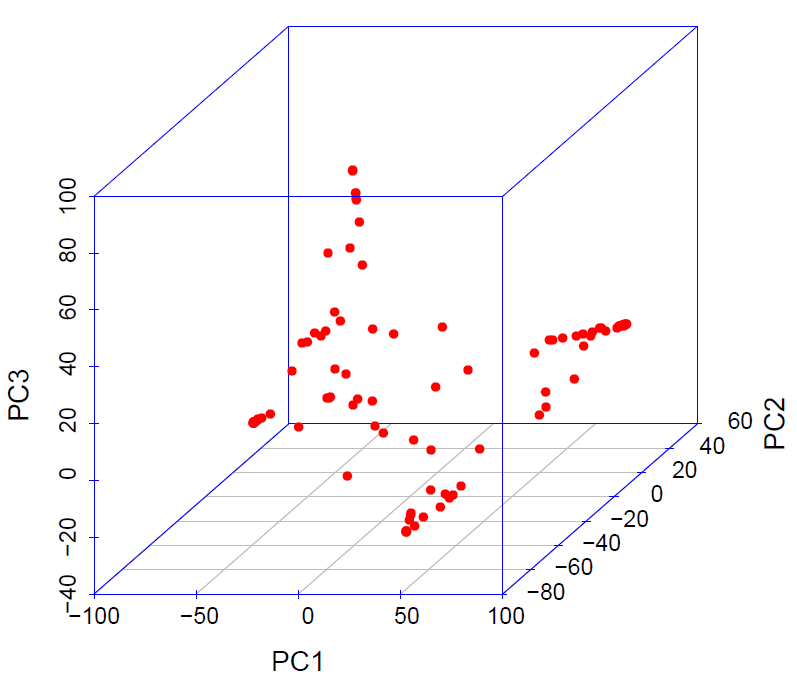


Fig S4. 3D plots of principal component of all 102 peanut accessions.
